# Tracking Turnover Among Health Care Workers During the COVID-19 Pandemic: A Cross-sectional Study

**DOI:** 10.1001/jamahealthforum.2022.0371

**Published:** 2022-04-08

**Authors:** Bianca K. Frogner, Janette S. Dill

**Affiliations:** 1 Center for Health Workforce Studies, Department of Family Medicine, School of Medicine, University of Washington, Seattle; 2 Division of Health Policy and Management, School of Public Health, University of Minnesota, Minneapolis

## Abstract

**Importance:**

The health care sector lost millions of workers during the COVID-19 pandemic and job recovery has been slow, particularly in long-term care.

**Objective:**

To identify which health care workers were at highest risk of exiting the health care workforce during the COVID-19 pandemic.

**Design, Setting, and Participants:**

This was an observational cross-sectional study conducted among individuals employed full-time in health care jobs from 2019 to 2021 in the US. Using the data from the Current Population Survey (CPS), we compared turnover rates before the pandemic (preperiod, January 2019-March 2020; 71 843 observations from CPS) with the first 9 months (postperiod 1, April 2020-December 2020; 38 556 observations) and latter 8 months of the pandemic (postperiod 2, January 2021-October 2021; 44 389 observations).

**Main Outcomes and Measures:**

Health care workforce exits (also referred to as turnover) defined as a health care worker's response to the CPS as being unemployed or out of the labor force in a month subsequent to a month when they reported being actively employed in the health care workforce. The probability of exiting the health care workforce was estimated using a logistic regression model controlling for health care occupation, health care setting, being female, having a child younger than 5 years old in the household, race and ethnicity, age and age squared, citizenship status, being married, having less than a bachelor’s degree, living in a metropolitan area, identifier for those reporting employment status at the first peak of COVID-19, and select interaction terms with time periods (postperiods 1 and 2). Data analyses were conducted from March 1, 2021, to January 31, 2022.

**Results:**

The study population comprised 125 717 unique health care workers with a mean (SD) age of 42.3 (12.1) years; 96 802 (77.0%) were women; 84 733 (67.4%) were White individuals. Estimated health care turnover rates peaked in postperiod 1, but largely recovered by postperiod 2, except for among long-term care workers and physicians. We found a 4-fold difference in turnover rates between physicians and health aides or assistants. Rates were also higher for health workers with young children (<5 years), for both sexes and highest among women. By race and ethnicity, persistently higher turnover rates were found among American Indian/Alaska Native/Pacific Islander workers; White workers had persistently lower rates; and Black and Latino workers experienced the slowest job recovery rates.

**Conclusions and Relevance:**

The findings of this observational cross-sectional study suggest that although much of the health care workforce is on track to recover to prepandemic turnover rates, these rates have been persistently high and slow to recover among long-term care workers, health aides and assistants, workers of minoritized racial and ethnic groups, and women with young children. Given the high demand for long-term care workers, targeted attention is needed to recruit job-seeking health care workers and to retain those currently in these jobs to lessen turnover.

## Key Points

**Question:**

Which health care workers were at highest risk of leaving the workforce during the COVID-19 pandemic compared with prepandemic levels?

**Findings:**

This observational cross-sectional study among 125 717 health care workers found that long-term care workers and physicians saw an upward trend in turnover rates. Health care workers employed as health aides and assistants, those of historically marginalized racial and ethnic groups, and those with young children, particularly women, had persistently high turnover rates and were experiencing a slow recovery.

**Meaning:**

These findings suggest that turnover rates are returning to prepandemic levels across most groups of health care workers, yet the recovery is uneven; targeted solutions are needed to ensure an adequate health care workforce is available to meet patient demand.

## Teaser

This observational cross-sectional study of health care workers in the US compares prepandemic and postpandemic rates of job turnover during the COVID-19 pandemic to understand who was at greatest risk of exiting the workforce.

## Introduction

Health care workers were deemed to be among the essential workers at the start of the COVID-19 pandemic.^1^ Despite calls to retrain and redeploy them, approximately 1.5 million health care workers lost their jobs during the first peak of the pandemic (April 2020) when clinics closed temporarily and hospitals postponed surgeries to prevent the spread of the SARS-CoV-2 virus.^2,3^ Although most of those jobs returned by the fall of 2020 and the job market continued to improve, as of November 2021 health care employment was still 2.7% lower than prepandemic levels .^4^ Despite an apparent overall recovery, skilled nursing facilities have experienced continual declines in employment levels since the start of the pandemic.^5^ Leaders in the field have highlighted concerns about the struggles facing the health care workforce, including burnout and a lack of available childcare, which may be contributing to shortages and putting patient care at risk.^6,7^

It has been reported that health care workers left their jobs because of the risk of getting COVID-19 and dying.^8^ In part, these concerns were in response to supply chain issues that challenged the timely distribution of personal protective equipment and a lack of COVID-19−related training.^9^ Long-term care (LTC) workers and health assistants and aides (including nursing assistants, home care aides, and medical assistants) have been highlighted throughout the pandemic as a particularly vulnerable group of employees. Too often, assistants and aides have no paid sick leave and/or adequate childcare, leaving them to choose between staying employed or caring for their personal health and the health of their families.^10,11,12^ Although emergency paid sick leave was offered under the Families First Coronavirus Response Act,^13^ workers in the health care sector were among the 25% who were ineligible for the benefit.^14^ The Federal Pandemic Unemployment Compensation program,^15^ which added $600 in weekly payment to state unemployment benefits through July 2020, raised concerns that workers may be enticed to remain unemployed rather than return to work; however, the evidence suggests that high unemployment rates have been driven by the loss of available jobs, childcare, and fear about safety in the workplace.^16^

During the course of the COVID-19 pandemic, health care job reports focused on the number of open and filled jobs rather than tracking which workers were leaving their jobs, either through unemployment (including resignations and terminations) and exiting the labor force.^17^ This study used data from a national household survey to identify which health care workers who lost or left their jobs during the COVID-19 pandemic, who was at highest risk of exiting the workforce, and whether they were currently searching for work. Survey data from the pandemic period were compared with prepandemic employment trends. Findings from this study will contribute to understanding of how COVID-19 has affected the US health care workforce during the 2 years of the pandemic and beyond. This study sets the stage for future workforce tracking, as well as for guiding the development of targeted policies to support retention and re-employment efforts.

## Methods

This observational cross-sectional study was exempted from institutional review because it used only a publicly available data set. The authors followed the Strengthening the Reporting of Observational Studies in Epidemiology (STROBE) reporting checklist for cross-sectional studies.

### Data Collection and Sample

We used the Current Population Survey (CPS), a US nationwide household survey conducted jointly by the US Census Bureau and the US Bureau of Labor Statistics (BLS), extracted from IPUMS (a public data service of the University of Minnesota).^18^ Each month, a series of labor force and demographic questions, known as the basic monthly survey, is administered. The CPS sample is representative of the civilian household-based population of the US. Each monthly CPS comprises 140 000 individuals living in approximately 70 000 households. When selected for the CPS sample, household members are surveyed in 4 consecutive months, left unenumerated during the subsequent 8 months, and then resurveyed in each of another 4 consecutive months; new rotation groups are brought into the CPS sample every calendar month.^19^

We used an enhanced version of the CPS that included harmonized measures from all basic monthly surveys, allowing us to identify changes in employment status for the same person across consecutive months.^20^ The panel nature of the basic monthly data has been largely untapped for the purposes of measuring unemployment among the health care workforce. We included observations in the CPS from January 2019 to October 2021. The analytical sample included individuals who were 18 to 65 years old and employed full-time. The study sample was composed of 71 843 observations in the prepandemic period from January 2019 to March 2020 (preperiod), 38 556 observations in the first 9 months of the pandemic (postperiod 1, April-December 2020), and 44 389 in the latter 8 months of the pandemic (postperiod 2, January-October 2021).

### Measures

The primary outcome was health care worker turnover, that is, exiting the workforce either through unemployment (ie, job losers or job leavers per BLS^18^) or exiting the labor force. We examined transitions to unemployment (job loss and job leaver) and leaving the labor force separately. We also examined transitions to unemployment and leaving the workforce separately. Transition to unemployment was identified when an individual who had reported being employed during the prior month’s survey reported being unemployed in the subsequent month. The survey defined unemployment as not currently working but being *available* to work and *searching* for a job during the prior 4 weeks or *waiting to be recalled* to a job from which they had been laid off. Leaving the labor force was identified when an individual reported being employed in the prior month and then reported not being employed during the survey reference week and *not actively looked* for work during the past 4 weeks. We did not include transitions to another job in our definition of turnover.

We included measures for an individual’s health care occupation and health care setting as key independent variables. We collapsed approximately 50 health care occupations as defined by the US Census Bureau into 8 categories based on similarities in title and educational requirements: (1) physicians; (2) advanced practitioners (excluding nurses); (3) registered nurses (RNs), including nurse practitioners; (4) therapists; (5) health care technicians (eg, ultrasonography and radiation technicians); (6) licensed practical nurses and licensed vocational nurses (LPNs/LVNs); (7) community-based workers; and (8) assistants and aides (eg, certified nursing assistants, home health aides; reference). Additional details are available in eTable 1 in the Supplement. We used 4 health care settings categories based on US Census Bureau industry codes: (1) hospitals (reference); (2) ambulatory care; (3) LTC, including home health; and (4) other health care settings. We lagged occupation and industry by 1 month so that the variable would indicate the occupation or industry in which a person worked before they exited the workforce.

The analyses models included several variables for sociodemographic information: binary measures for being female, age and age squared, race and ethnicity, citizenship status, being married, residing in a metropolitan area, having less than a college degree, and having a child younger than 5 years of age living in the household. We created the following mutually exclusive race and/or ethnicity categories: American Indian/Alaska Native/Pacific Islander, Asian, Black, Latino, White (reference), and multiple race or other race or ethnicity. We had a categorical variable for citizenship status: born in the US (reference), naturalized US citizen, or not a US citizen.

Research has indicated that, compared with parents of older children, parents of younger children struggled to maintain employment during the COVID-19 pandemic.^21^ We tested whether having school-aged children in the household was associated with job status and found that it was not a significant factor among health care workers; consequently, we chose to focus on younger children. We included an interaction between sex and parenthood status defined as having a child younger than 5 years old to test whether mothers were more likely to turn over.

We included a binary measure for whether an individual reported turnover in April 2020, to reflect the economy-wide drop in employment brought on by the initial wave of the pandemic. Finally, all analyses were weighted to ensure that the sample was representative of the US population.

### Statistical Analyses

Logit models were used to estimate whether a health care worker would turn over. To compare employment changes over time, we interacted select characteristics with a 3-category variable indicating whether the individual was reporting in the preperiod, postperiod 1, or postperiod 2, and reported the estimated probability of turnover for each of these interactions. We also ran separate models to estimate the likelihood of becoming unemployed or leaving the labor force to identify any driving factors. Separate models were run for interactions of sex or parenthood, race and/or ethnicity, health care setting, and occupation. We presented the estimated probability of turnover by sex and parenthood, race and ethnicity, health care setting, and occupation. The full models used for calculating the estimated probabilities are available in eTables 2 to 5 in the Supplement.

Data analyses were conducted from March 1, 2021, to January 31, 2022, using Stata, version 17.0 (StataCorp LLC). Statistical significance was defined as *P* < .001.

## Results

**Figure 1.**
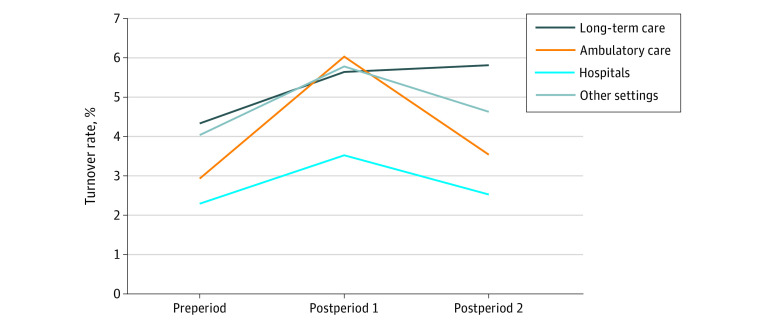
Estimated Turnover Rates of Health Care Workers, by Health Care Setting, 2019 to 2021 Preperiod, January 2019-March 2020; postperiod 1, April-December 2020; postperiod 2, January-October 2021; values for “other” health care settings are available in eTable 1 in the Supplement; regression models used for calculating estimated leaver status are shown in eTable 2 in the Supplement.

**Figure 2.**
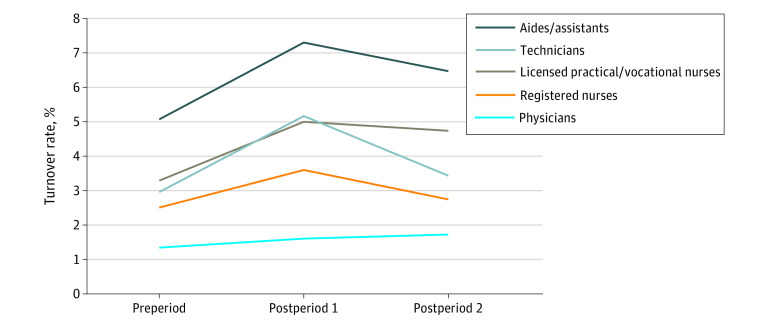
Estimated Turnover Rates of Health Care Workers, by Health Care Occupation, 2019 to 2021 Preperiod, January 2019-March 2020; postperiod 1, April-December 2020; postperiod 2, January-October 2021; registered nurses include advanced practice registered nurses; other advanced practitioners, therapists, and community-based workers omitted from figure with values available in the Supplement; regression models used for calculating estimated leaver status are shown in eTable 3 in the Supplement.

The study population comprised CPS responses for 125 717 unique health care workers (mean [SD] age, 42.3 [12.1] years; 98 802 [77.0%] women; 1760 [1.4%] American Indian/Alaska Native/Pacific Islander; 8423 [6.7%] Asian; 16 343 [13.0%] Black; 12 697 [10.1%] Latino; 84 733 [67.4%] White; and 1634 [1.3%] individuals of multiple/other races and/or ethnicities). In the preperiod (January 2019-March 2020), an average of 3.2% health care workers reported turnover; in postperiod 1 (April-December 2020), 5.6%; and in postperiod 2 (January-October 2021), 3.7%. Except for among LTC workers (Figure 1) and physicians (Figure 2), turnover rates were highest in postperiod 1, with postperiod 2 rates declining to slightly higher than preperiod rates across all sociodemographic groups (ie, sex/parenthood status, race/ethnicity, setting, and occupation). Labor force exits exceeded unemployment rates for every group throughout the study period, except in postperiod 2 among individuals who reported being of multiple races or of other race and/or ethnicity (more details are available in eTable 6 in the Supplement). Generally, unemployment contributed more to the overall turnover rates in the postperiods than in the preperiod, with workers in postperiod 1 more likely associated with exiting the labor force than to unemployment.

Throughout the study period, health care workers in hospitals were less likely to turn over than workers in other settings, with rates in postperiod 2 slightly higher than in the preperiod (Figure 1). Ambulatory care workers had more than double the turnover rate in postperiod 1 compared with the preperiod. Despite considerable recovery by postperiod 2, rates remained 0.7 percentage points higher than the preperiod. Turnover rates were highest for LTC workers in the preperiod and continued to increase over time.

Turnover rates varied widely across health care occupations (Figure 2), with a nearly 4-fold difference between jobs associated with lower wages (eg, aides/assistants) compared with higher wages (eg, physicians). Health aides and assistants had the highest turnover rates throughout the study period and the rate remained at 1.3 percentage points higher than in the preperiod. Notably, turnover rates among LPNs/LVNs and technicians experienced similar increases from the preperiod to postperiod 1, but LPNs/LVNs experienced a slower recovery of jobs in postperiod 2, with rates remaining 1.4 percentage points higher than in the preperiod. Despite low overall turnover rates, physicians were the only occupational group to see continuous turnover increases over time.

**Figure 3.**
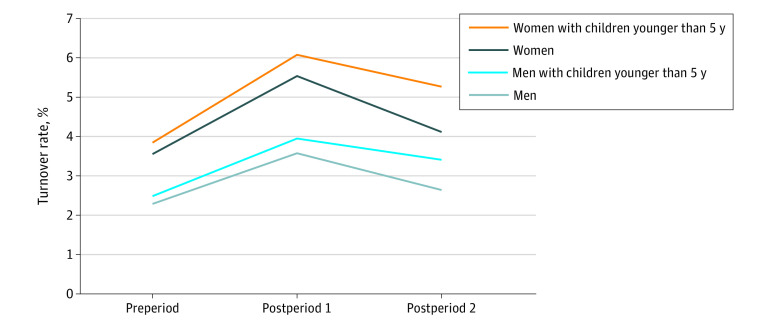
Estimated Turnover Rates of Health Care Workers, by Sex and Having Children Younger Than 5 Years Old, 2019 to 2021 Preperiod, January 2019-March 2020; postperiod 1, April-December 2020; postperiod 2, January-October 2021; regression models used for calculating estimated leaver status are shown in eTable 4 in the Supplement.

Women in health care were consistently more likely to turn over compared with men across all time periods (Figure 3). Health care workers of both sexes with young children were more likely to turn over, and experienced slower recovery in postperiod 2, than the group without young children in the household. Turnover rates were highest among women with young children from among all sex and parenthood groups.

**Figure 4.**
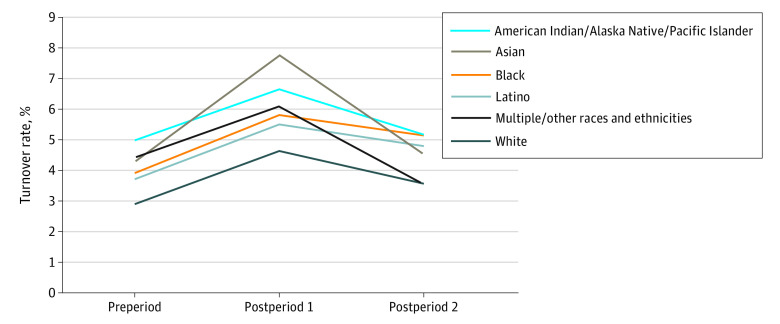
Estimated Turnover Rates of Health Care Workers, by Race and Ethnicity, 2019 to 2021 Estimated probabilities for American Indian/Alaska Native/Pacific Islanders and Black individuals are nearly equivalent at 1 decimal place in postperiod 2 (overlap); preperiod, January 2019-March 2020; postperiod 1, April-December 2020; postperiod 2, January-October 2021; regression models for estimated status of those exiting the workforce are shown in eTable 5 in the Supplement.

In the preperiod and in postperiod 1, the lowest turnover rates were among White health care workers; in preperiod 2, the lowest rates were among health care workers who reported being of multiple or other race and/or ethnicity, just ahead of White health care workers (Figure 4). As mentioned, health care workers self-identifying as being of multiple or other races or ethnicities were the only group among which rates improved in the postperiod 2 compared with the preperiod. This finding may have been influenced by the small sample of respondents in this category. Throughout the study period, turnover rates among health care workers identifying as American Indian/Alaska Native/Pacific Islander were persistently high compared with those of the other racial or ethnicity categories. An exception was the finding among Asian health care workers—the race category with the highest exit rates in postperiod 1. Rates among Asian health care workers experienced the sharpest increase between preperiod and postperiod 1, but largely recovered by postperiod 2. Black and Latino health care workers followed a similar pattern over time, with rates among both groups experiencing a slow recovery in postperiod 2.

## Discussion

Employment turnover among nearly all segments of the health care workforce has not yet fully recovered from the COVID-19 pandemic, with turnover rates among LTC workers and physicians worsening over time. While these trends are consistent with recent reports on employment in health care,^4,5^ to our knowledge, this study provides a more comprehensive and robust analysis of health employment transitions over a longer time horizon and after the peak of the pandemic.

Turnover rates were mostly driven by exits from the labor force rather than unemployment, despite unemployment being a slightly larger driver in the postperiods than in the preperiod. The underlying reasons for why an individual becomes unemployed vs leaving the labor force warrants attention. Being unemployed suggests that individuals are still seeking work, while those who exit the labor force are not, likely because of the availability of other resources (eg, working spouse) to sustain household income. The higher unemployment rates in postperiod 2 than in the preperiod signals a constraint on available jobs, but what is unknown is the extent to which workers were choosing to stay unemployed until they could find a job with acceptable conditions (eg, wages or safety precautions) or for which they were qualified. For those exiting the labor force, further work is needed to understand whether workers are involuntarily leaving their jobs and may be marginally attached workers who would like a job only if the circumstances are right.^22^

The highest turnover rates were among health aides and assistants compared with all other health care occupations, followed by LPNs/LVNs who experienced a slow recovery in postperiod 2. These patterns were likely to be associated with turnover rates in the LTC setting (which we were unable to test because of collinearity in the model), a common employer of LPNs/LVNs and heavily reliant on health aides and assistants (eg, nursing assistants and home health aides).^23,24^ The turnover patterns we found in the LTC setting support the findings of a study conducted earlier in the COVID-19 pandemic,^25^ which reported that 1 in 5 skilled nursing facilities had staffing shortages, with a high demand for nursing assistants and nurses. The shortages may have been partly explained by the lack of available personal protective equipment (eg, N95 masks, gowns).^25^Another study found that staffing hours in nursing homes did not decline substantially despite the staffing shortages; the researchers were not able to identify how the hours remained steady despite fewer workers.^26^

Similar to findings that the BLS reported using CPS data,^27^ our study sample of women working in health care with children younger than 5 years old had the highest probability of leaving their jobs. Another recent study found a substantial gender gap in employment among those with young children (<5 years), although it was not the largest gap when compared with parents of older children.^28^ Although the gender gap in employment between those with young children (<5 years) was not the largest gap when comparing with parents of older children, women with young children were most likely to exit the labor force. Our study findings support this statement, and other past research has also found marked reductions in labor market activity among mothers of young children during the pandemic.^21,29,30,31^

We found that health care workers from historically marginalized racial and ethnic groups had higher turnover rates compared with White health care workers. Although we did not look at the interactions among sex, race and ethnicity, and occupation or sector, previous studies have documented that women of historically marginalized racial and ethnic groups are heavily represented in lower-skilled jobs (defined by educational requirements), such as health assistant and aide positions, many of which are found in the LTC setting.^32,33^ The COVID-19 pandemic has put on full display the inequities of job security and of benefits available to workers of racial and ethnic minority groups, combined with the unbalanced community exposure to and treatment of COVID-19—all of which are factors that put employment at risk.^34,35,36^

### Limitations

Our study had a number of limitations. First, we did not determine whether exiting the workforce was associated with COVID-19. Although the CPS did add questions on the role that COVID-19 played in an individual’s ability to work, the questions were added to IPUMS late in our analysis. Data summarized by BLS suggest that workers were more likely to report loss of employment associated with COVID-19 in December 2020 than in December 2021, and that those in health care support occupations, such as aides and assistants, experienced the consequences of COVID-19 workforce changes more so than did other health care workers.^37^ Second, we did not examine wages and the role of unemployment benefits that could be associated with transitions to unemployment and/or exiting the labor force; the CPS collects only limited wage information. Some evidence suggests that wages rose in response to low workforce supply to attract much-needed workers.^5,38^ Third, we did not look at re-employment or entry of new workers. A recent study of direct care workers (which overlaps with the aides/assistants category) found that only two-thirds of workers displaced by consequences of COVID-19 returned to direct care work by the first quarter of 2021 and found no evidence of large scale movement of displaced workers from the retail or hospitality sectors, common sources of employment in prepandemic times.^38,39^ Fourth, CPS response rates were considerably lower in April to June 2021, with in-person interviews halted, which lead to concerns about whether unemployment estimates were being underestimated; however, but the BLS did not find evidence of systematic bias.^40^ Still, workforce turnover may have been even higher in postperiod 1 given that we found a misclassification of temporarily laid-off workers as employed.^41^ Lastly, we did not consider policy changes in vaccine mandates or masking requirements, which often were instituted by state but also by employers; these were not identifiable in the CPS data. Further study is needed to understand the role of vaccine mandates, schools reopenings, and burnout on the short-term and long-term trajectory of job loss and resignation in the health care workforce.

## Conclusions

The findings of this national cross-sectional study suggest that, despite continued elevated turnover rates among health care workers since the start of the COVID-19 pandemic, many groups are on track to recover. This study does not suggest mass exits by any particular profession, although growing turnover rates among physicians do support concerns about burnout.^42,43^ Long-term care workers warrant attention given their persistently high and increasing exit rates. Health aides and assistants and LPNs/LVNs may be key groups for targeted attention, particularly if they work in LTC where they remain in high demand.^44^ As numerous media headlines have pointed out, women, particularly those with young children, have experienced the largest share of the COVID-19−related job losses. Health care, a field that is predominately composed of women, many of whom are of historically marginalized racial and ethnic groups, is no different.

Although these study findings suggest that the large fluctuations in workforce turnover witnessed earlier in the pandemic have been tempered in the latter months, COVID-19 continues to persist in the community with the emergence of new variants and because of the challenges with vaccinating the global population. With ongoing concerns about burnout leading to early retirement, particularly among nurses, continuing to track turnover among health care workers will be critical to determining our future focus, whether it should be on job placement, retention, or quality (eg, higher wages, improved benefits). In addition, it is critical that we distinguish between terminations, resignations, and those exiting the labor force. Waiting too long to understand these issues may further elongate the consequences of the COVID-19 pandemic.

## Notes

Supplement. **eTable 1. **Crosswalk of detailed health care occupation codes and occupation groups used in study **eTable 2.** Logit model estimating turnover rates among health care workers by health care sector, 2019-2021 **eTable 3.** Logit model of estimating turnover rates among health care workers by health care occupation, 2019-2021 **eTable 4.** Logit model of estimating turnover rates among health care workers by gender and parenthood status, 2019-2021 **eTable 5.** Logit model of estimating turnover rates among health care workers by race-ethnicity, 2019-2021 **eTable 6. **Estimated probability of employment status by sociodemographic characteristics
