# Supplementary material for: Tracking Turnover Among Health Care Workers During the COVID-19 Pandemic: A Cross-sectional Study
Source: JAMA Health Forum. 2022 Apr 8;3(4):e220371. doi: 10.1001/jamahealthforum.2022.0371 (PMC8994131; doi:10.1001/jamahealthforum.2022.0371)
Supplement: Supplement. — eTable 1. Crosswalk of detailed health care occupation codes and occupation groups used in study eTable 2. Logit model estimating turnover rates among health care workers by health care sector, 2019-2021 eTable 3. Logit model of estimating turnover rates among health care workers by health care occupation, 2019-2021 eTable 4. Logit model of estimating turnover rates among health care workers by gender and parenthood status, 2019-2021 eTable 5. Logit model of estimating turnover rates among health care workers by race-ethnicity, 2019-2021 eTable 6. Estimated probability of employment status by sociodemographic characteristics [file jamahealthforum-e220371-s001.pdf]

Supplemental Online Content

Frogner BK, Dill JS. Tracking turnover among health care workers during the COVID-19 pandemic: a cross-sectional study. *JAMA Health Forum.* 2022;3(4):e220371. doi:10.1001/jamahealthforum.2022.0371

eTable 1. Crosswalk of detailed health care occupation codes and occupation groups used in study

eTable 2. Logit model estimating turnover rates among health care workers by health care sector, 2019-2021

eTable 3. Logit model of estimating turnover rates among health care workers by health care occupation, 2019-2021

eTable 4. Logit model of estimating turnover rates among health care workers by gender and parenthood status, 2019-2021

eTable 5. Logit model of estimating turnover rates among health care workers by race-ethnicity, 2019-2021

eTable 6. Estimated probability of employment status by sociodemographic characteristics

This supplemental material has been provided by the authors to give readers additional information about their work.

© 2022 Frogner BK et al. *JAMA Health Forum.*

eTable 1. Crosswalk of Detailed Health Care Occupation Codes and Occupation Groups Used in Study

Census Occupation Code	Census Job Title	Occupation Group Used in Study
0350	Medical and health services managers	Community-Based Workers
0420	Social and community service managers	Community-Based Workers
1650	Medical scientists	Advanced Practitioners
1820	Psychologists	Advanced Practitioners
2000	Counselors	Community-Based Workers
2010	Social workers	Community-Based Workers
2025	Miscellaneous community and social service specialists, including health educators and community health workers	Community-Based Workers
3000	Chiropractors	Advanced Practitioners
3010	Dentists	Advanced Practitioners
3030	Dietitians and nutritionists	Advanced Practitioners
3040	Optometrists	Advanced Practitioners
3050	Pharmacists	Advanced Practitioners
3060	Physicians and surgeons	Physicians
3110	Physician assistants	Advanced Practitioners
3120	Podiatrists	Advanced Practitioners
3140	Audiologists	Advanced Practitioners
3150	Occupational therapists	Advanced Practitioners
3160	Physical therapists	Advanced Practitioners
3200	Radiation therapists	Therapists
3210	Recreational therapists	Therapists
3220	Respiratory therapists	Therapists
3230	Speech-language pathologists	Advanced Practitioners
3235	Exercise physiologists	Therapists
3245	Exercise physiologists and therapists, all other	Therapists
3255	Registered nurses	RNs
3256	Nurse anesthetists	APRNs
3257	Nurse midwives	APRNs
3258	Nurse practitioners	APRNs
3260	Health diagnosing and treating practitioners, all other	Advanced Practitioners
3300	Clinical laboratory technologists and technicians	Technicians
3310	Dental hygienists	Technicians
3320	Diagnostic related technologists and technicians	Technicians
3400	Emergency medical technicians and paramedics	Technicians
3420	Health diagnosing and treating practitioner support technicians	Technicians
3500	Licensed practical and licensed vocational nurses	LPNs/LVNs
3510	Medical records and health information technicians	Technicians
3520	Opticians, dispensing	Technicians
3535	Miscellaneous health technologists and technicians	Technicians
3540	Other healthcare practitioners and technical occupations	Technicians
3600	Nursing, psychiatric, and home health aides	Aides/Assistants
3610	Occupational therapist assistants and aides	Therapists
3620	Physical therapist assistants and aides	Therapists
3630	Massage therapists	Therapists
3640	Dental assistants	Aides/Assistants
3645	Medical assistants	Aides/Assistants

© 2022 Frogner BK et al. *JAMA Health Forum.*

3646	Medical transcriptionists	Aides/Assistants
3647	Pharmacy aides	Aides/Assistants
3649	Phlebotomists	Aides/Assistants
3655	Miscellaneous healthcare support occupations, including medical equipment preparers	Aides/Assistants
4610	Personal and home care aides	Aides/Assistants
8760	Medical, dental, and ophthalmic laboratory technicians	Technicians
9110	Ambulance drivers and attendants, except emergency medical technicians	Aides/Assistants

Source: Occupation codes: 2011-2019 (2010 Census classification scheme. IPUMS CPS. Accessed August 21, 2020. https://cps.ipums.org/cps/codes/occ_20112019_codes.shtml

© 2022 Frogner BK et al. *JAMA Health Forum.*

eTable 2. Logit model estimating turnover rates among health care workers by health care sector, 2019-2021

	Coefficient	Std. err.
Pre-Period	Ref	Ref
Post-Period 1	0.462***	0.075
Post-Period 2	0.111	0.081
**Setting**		
Hospital	Ref	Ref
Ambulatory	0.252***	0.074
LTC	0.671***	0.074
Other settings	0.592***	0.069
**Interaction terms**		
Post-Period 1#Hospital	Ref	Ref
Post-Period 1#Ambulatory	0.317**	0.101
Post-Period 1#LTC	-0.175	0.105
Post-Period 1#Other settings	-0.066	0.096
Post-Period 2#Hospital	Ref	Ref
Post-Period 2#Ambulatory	0.102	0.112
Post-Period 2#LTC	0.209	0.110
Post-Period 2#Other settings	0.044	0.104
**Sex/parenthood**		
Female	0.333***	0.040
Children less than 5y	0.140***	0.043
**Race-ethnicity**		
White	Ref	Ref
Black	0.264***	0.043
Latino	0.228***	0.046
Asian	0.442***	0.064
American Indian/Alaska Native/Pacific Islander	0.4104***	0.105
Multiple race categories or other	0.250*	0.113
**Other demographic variables**		
US born	Ref	Ref
Naturalized citizen	0.053	0.056
Not citizen	0.292***	0.067
Married	-0.111***	0.033
Age	-0.151***	0.008
Age squared	0.002***	0.000
Less than a college degree	0.650***	0.032
Metropolitan area	0.188***	0.041
April 2020 dummy variable	1.070***	0.056
Constant	-1.000***	0.174
Pseudo R2	0.0648	
Observations	125,717	

*** p<0.01, ** p<0.05, * p<0.1

© 2022 Frogner BK et al. *JAMA Health Forum.*

eTable 3. Logit model of estimating turnover rates among health care workers by health care occupation, 2019-2021

	Coefficient	Std. err.
Pre-Period	Ref	Ref
Post-Period 1	0.398***	0.055
Post-Period 2	0.252***	0.055
**Occupations**		
Physicians	-1.407***	0.186
Advanced Practitioners	-0.615***	0.108
RNs	-0.750***	0.078
Therapists	-0.593***	0.145
Techs	-0.576***	0.082
LPN/LVN	-0.470***	0.123
Aides/asst	Ref	Ref
Community-based workers	-0.758***	0.084
**Interaction terms**		
Post-period 1#Physicians	-0.205	0.269
Post-period 1#Advanced Practitioners	0.262	0.138
Post-period 1#RNs	-0.012	0.105
Post-period 1#Therapists	0.805***	0.180
Post-period 1#Technicians	0.193	0.112
Post-period 1#LPN/LVN	0.058	0.177
Post-period 1#Aides/assistants	Ref	Ref
Post-period 1#Community-based workers	0.060	0.112
Post-period 2#Physicians	0.018	0.266
Post-period 2#Advanced Practitioners	-0.235	0.157
Post-period 2#RNs	-0.171	0.113
Post-period 2#Therapists	0.237	0.203
Post-period 2#Technicians	-0.110	0.122
Post-period 2#LPN/LVN	0.140	0.183
Post-period 2#Aides/assistant	Ref	Ref
Post-period 2#Community-based workers	-0.134	0.119
**Sex/parenthood**		
Female	0.261***	0.041
Children less than 5y	0.138**	0.043
**Race-ethnicity**		
White	Ref	Ref
Black	0.232***	0.043
Latino	0.179***	0.047
Asian	0.419***	0.065
American Indian/Alaska Native/Pacific Islander	0.426***	0.105
Multiple race categories or other	0.275*	0.114
**Other demographic variables**		
US born	Ref	Ref
Naturalized citizen	-0.002	0.057

© 2022 Frogner BK et al. *JAMA Health Forum.*

Not citizen	0.225***	0.068
Married	-0.089**	0.033
Age	-0.138***	0.008
Age squared	0.001***	0.000
Less than a college degree	0.347***	0.039
Metropolitan area	0.179***	0.041
April 2020 dummy variable	1.076***	0.058
Constant	-0.632***	0.171
Pseudo R2	0.0692	
Observations	125,717	

*** p<0.01, ** p<0.05, * p<0.1

RN=registered nurse including advanced practice registered nurse; LPN/LVN = licensed practical nurse/licensed vocational nurse

© 2022 Frogner BK et al. *JAMA Health Forum.*

eTable 4. Logit model of estimating turnover rates among health care workers by gender and parenthood status, 2019-2021

	Coefficient	Std. err.
Pre-Period	Ref	Ref
Post-Period 1	0.479***	0.040
Post-Period 2	0.168***	0.040
**Parenthood**		
Child less than 5	0.090	0.069
**Interaction terms**		
Post-period 1#Child less than 5	0.009	0.096
Post-period 2#Child less than 5	0.171	0.100
**Sex**		
Female	0.341***	0.040
**Race-ethnicity**		
White	Ref	Ref
Black	0.315***	0.042
Latino	0.253***	0.046
Asian	0.415***	0.064
American Indian/Alaska native/ Pacific Islander	0.457***	0.105
Multiple race categories or other	0.270*	0.113
**Other demographic variables**		
US born	Ref	Ref
Naturalized citizen	0.061	0.057
Not citizen	0.357***	0.067
Married	-0.145***	0.033
Age	-0.154***	0.008
Age squared	0.002***	0.000
Less than a college degree	0.712***	0.031
Metropolitan area	0.157***	0.041
April 2020 dummy variable	1.072***	0.057
Constant	-1.012***	0.168
Pseudo R2	0.0570	
Observations	125,717	

*** p<0.01, ** p<0.05, * p<0.1

© 2022 Frogner BK et al. *JAMA Health Forum.*

eTable 5. Logit model of estimating turnover rates among health care workers by race-ethnicity, 2019-2021

	Coefficient	Std. err.
Pre-Period	Ref	Ref
Post-Period 1	0.495***	0.047
Post-Period 2	0.192***	0.049
**Race-ethnicity**		
White	Ref	Ref
Black	0.307***	0.068
Latino	0.256***	0.074
Asian	0.407***	0.102
American Indian/Alaska Native/Pacific Islander	0.563***	0.165
Multiple race categories or other	0.439**	0.176
**Interaction Terms**		
Post-Period 1#White	Ref	Ref
Post-Period 1#Black	-0.060	0.095
Post-Period 1#Latino	-0.072	0.104
Post-Period 1#Asian	0.150	0.132
Post-Period 1# American Indian/Alaska Native/Pacific Islander	-0.175	0.240
Post-Period 1# Multiple race categories or other	-0.141	0.255
Post-Period 2#White	Ref	Ref
Post-Period 2#Black	0.097	0.100
Post-Period 2#Latino	0.069	0.107
Post-Period 2#Asian	-0.169	0.147
Post-Period 2# American Indian/Alaska Native/Pacific Islander	-0.162	0.268
Post-Period 2# Multiple race categories or other	-0.463	0.296
**Sex/parenthood**		
Female	0.342***	0.040
Children less than 5y	0.144***	0.043
**Other demographic variables**		
US born	Ref	Ref
Naturalized citizen	0.060	0.057
Not citizen	0.358***	0.067
Married	-0.145***	0.033
Age	-0.154***	0.008
Age squared	0.002***	0.000
Less than a college degree	0.712***	0.031
Metropolitan area	0.158***	0.041
April 2020 dummy variable	1.071***	0.057
Constant	-1.028***	0.168
Pseudo R2	0.0574	
Observations	125,717	

*** p<0.01, ** p<0.05, * p<0.1

© 2022 Frogner BK et al. *JAMA Health Forum.*

eTable 6. Estimated probability of employment status by sociodemographic characteristics

	Unemployed			Exited Labor Force			Either Unemployed or Exited Labor Force		
	Pre-Period	Post-Period 1	Post-Period 2	Pre-Period	Post-Period 1	Post-Period 2	Pre-Period	Post-Period 1	Post-Period 2
**Setting**									
Hospitals	0.4%	1.2%	0.5%	1.9%	2.5%	2.0%	2.3%	3.6%	2.5%
Ambulatory settings	0.6%	3.0%	0.9%	2.3%	3.1%	2.7%	2.9%	6.1%	3.6%
Long-term care	1.2%	1.9%	1.9%	3.1%	3.9%	4.0%	4.3%	5.7%	5.8%
Other settings	0.9%	2.5%	1.4%	3.1%	3.4%	3.3%	4.0%	5.8%	4.7%
**Occupation**									
Physicians	0.2%	0.5%	0.3%	1.1%	1.1%	1.4%	1.3%	1.6%	1.7%
Advanced Practitioners	0.7%	2.4%	0.8%	2.1%	2.9%	2.1%	2.8%	5.3%	2.9%
Registered nurses including advanced practice registered nurses	0.5%	1.4%	0.8%	2.0%	2.3%	1.9%	2.5%	3.6%	2.7%
Therapists	0.4%	3.7%	1.5%	2.5%	5.4%	3.1%	2.9%	8.9%	4.6%
Technicians	0.5%	2.4%	0.9%	2.4%	2.7%	2.5%	3.0%	5.2%	3.4%
Licensed practical/vocational nurses	0.9%	1.8%	1.2%	2.4%	3.5%	3.5%	3.3%	5.0%	4.7%
Aides and assistants	1.2%	2.8%	1.8%	3.9%	4.7%	4.7%	5.1%	7.4%	6.4%
Community-based workers	0.7%	1.9%	0.8%	1.8%	2.0%	2.0%	2.5%	3.8%	2.8%
**Sex/Parenthood**									
Women	0.8%	2.3%	1.1%	2.7%	3.3%	3.0%	3.5%	5.5%	4.1%
Women with children under 5y	0.9%	2.2%	1.4%	2.9%	4.1%	3.9%	3.9%	6.1%	5.3%
Men	0.5%	1.5%	0.8%	1.7%	2.1%	1.9%	2.3%	3.6%	2.7%
Men with children under 5y	0.6%	1.5%	0.9%	1.9%	2.6%	2.5%	2.5%	3.9%	3.4%
**Race-ethnicity**									
White	0.7%	2.0%	0.9%	2.2%	2.7%	2.6%	2.9%	4.6%	3.5%
Black	0.8%	2.2%	1.5%	3.0%	3.7%	3.7%	3.9%	5.8%	5.1%
Latino	0.8%	2.1%	1.5%	2.9%	3.6%	3.3%	3.7%	5.5%	4.7%
Asian	0.5%	2.6%	1.1%	3.7%	5.5%	3.3%	4.3%	7.7%	4.4%
American Indian/Alaska Native/Pacific Islander	1.5%	2.3%	0.9%	3.5%	4.5%	4.2%	5.0%	6.6%	5.1%
Multiple race/ethnicity categories or other	1.3%	2.7%	1.8%	3.1%	3.4%	1.7%	4.4%	6.1%	3.4%

© 2022 Frogner BK et al. *JAMA Health Forum.*

Notes: Pre-Period=Jan 2019 to Mar 2020; Post-Period 1=Apr 2020 to Dec 2020; Post-Period 2=Jan 2021 to Oct 2021

© 2022 Frogner BK et al. *JAMA Health Forum.*
